# MicroRNAs as biomarkers for trastuzumab-based therapy in HER2-positive advanced oesophago-gastric cancer patients

**DOI:** 10.3389/fonc.2023.1258365

**Published:** 2023-11-29

**Authors:** Hazel Lote, Florentia Mousoullou, George Vlachogiannis, Andrea Lampis, Laura Satchwell, Clare Peckitt, Caroline Fong, Ruwaida Begum, Shannon Kidd, Susan Cromarty, Anderley Gordon, Charlotte Fribbens, Sheela Rao, Naureen Starling, Ian Chau, David Cunningham, Nicola Valeri

**Affiliations:** ^1^ Division of Molecular Pathology, The Institute of Cancer Research, London, United Kingdom; ^2^ Department of Gastrointestinal Oncology, The Royal Marsden Hospital NHS Foundation Trust, London, United Kingdom; ^3^ Department of Surgery and Cancer, Imperial College London, Hammersmith Hospital, London, United Kingdom

**Keywords:** oesophageal adenocarcinoma, gastric cancer, microRNA, HER2, biomarkers, trastuzumab

## Abstract

**Background:**

This study aimed to identify microRNAs (miRs) as circulating biomarkers of resistance to first-line trastuzumab-based therapy in advanced HER2-positive oesophago-gastric cancer patients.

**Methods:**

A high-throughput 1015 Exiqon miRCURY LNA™ microRNA inhibitor library screen was performed in trastuzumab-treated HER2-positive NCI-N87 and HER2-negative FLO-1 oesophago-gastric cancer cell lines. NanoString nCounter^®^ miR analysis was performed in NCI-N87, FLO-1, and MAGIC trial (ISRCTN93793971) formalin-fixed paraffin-embedded (FFPE) oesophago-gastric cancer patient samples. MiR-148a-3p copies in plasma samples were quantified using digital droplet polymerase chain reaction (ddPCR) from HER2-positive oesophago-gastric cancer patients treated with standard-of-care trastuzumab-based therapy within the FOrMAT (NCT02112357) and PLATFORM (NCT02678182) clinical trials. The primary endpoints were overall survival (OS) for plasma miR-148a-3p HIGH (>median) versus LOW (≤median). The secondary endpoints were progression-free survival (PFS) and 3-month progression-free rates (PFRs) miR-148a-3p HIGH versus LOW. PLATFORM sensitivity analysis normalised miR-148a-3p (NmiR-148a-3p).

**Results:**

The inhibition of miR-148a-3p reduced NCI-N87 relative cell viability (<0.6) and expression was high (>242) in NCI-N87 and HER2-positive MAGIC trial patients (n=5). Normalised-miR-148a-3p (NmiR-148a-3p) LOW versus HIGH demonstrated a statistically significant difference in 3-month PFRs (n=23; OR, 0.11 [0.02–0.78]; p=0.027; aOR, 0.03 [0.001–0.71], p=0.029) but no difference in OS or PFS. There was no statistically significant relationship between miR-148-3p LOW versus HIGH for OS (PLATFORM, n=62; hazard ratio [HR], 0.98 [0.57–1.66]; p=0.933; FOrMAT, n=8; HR, 0.54 [0.13–2.31]; p=0.322), PFS (n=62; HR, 1.08 [0.65–1.81]; p=0.759; FOrMAT, n=8; HR, 1.26 [0.31–5.07]; p=0.714), or PFRs (PLATFORM, n=31; odds ratio [OR], 0.67 [0.2–2.8]; p=0.577).

**Conclusion:**

Normalised miR-148a-3p may be a relevant biomarker for trastuzumab-based therapy in advanced HER2-positive oesophago-gastric cancer patients.

## Introduction

Oesophago-gastric cancer is the sixth most common cancer in Europe and the third most common cause of cancer death worldwide (1–3). Human epidermal growth factor receptor (HER2) positivity is defined as overexpression of the HER2 receptor, or HER2 gene amplification, and occurs in approximately 20% of oesophago-gastric cancer patients (4, 5). Among HER2-positive oesophago-gastric cancer patients treated with first-line standard-of-care trastuzumab-based chemotherapy, response rates were 47% and median overall survival (OS) was 13.8 months in the landmark phase 3 ToGA trial (4). Survival is limited by trastuzumab resistance. The identification of biomarkers might allow us to stratify patients, minimise unnecessary toxicity, and develop strategies to overcome resistance.

HER2 is a member of the epidermal growth factor receptor family (EGFR) and is a type I transmembrane tyrosine kinase growth factor receptor 1,255 amino acids in length, encoded by erb-B2 receptor tyrosine kinase 2 *(ERBB2)* on chromosome 17q21 (6, 7). HER2 is an orphan receptor with no natural ligand and no ligand-binding activity (8). HER2 exists in a constitutively activated conformation and activates downstream signalling via dimerisation with other members of the EGFR family (7, 9–11). When HER2 is overexpressed, the formation of HER2-HER2 homodimers leads to ligand-independent downstream signalling (7, 12–14). HER2 activates intracellular signalling pathways, including rat sarcoma (RAS)/rapidly accelerated fibrosarcoma (RAF)/mitogen-activated protein kinase kinase (MEK)/mitogen-activated protein kinase (MAPK)/myelocytomatosis oncogene (MYC)/c-jun, HER2/EGFR, HER2/HER3, Akt-mammalian target of rapamycin (mTOR), phosphatidylinositol-3-kinase (PI3K), and cyclin-D-cyclin-dependent protein kinase (CDK) complexes (7, 15–21). These pathways can affect cell proliferation, apoptosis, adhesion, migration, and differentiation (7, 15–21).

Trastuzumab is a humanised monoclonal antibody against HER2, with murine HER2-antigen-binding loops attached to the human IgG constant domain (22). Trastuzumab binds to the C-terminal portion of domain IV on the HER2 receptor, preventing heterodimer formation, blocking HER2 signalling, and engaging the endocytic removal of HER2 from the cell surface (11, 14, 23). Trastuzumab preferentially targets HER2 homodimers (24) and inhibits the proliferation of human tumour cells overexpressing HER2 (22). Mechanisms of resistance to trastuzumab include HER2 receptor downregulation due to selection pressure, with evidence that 35% of HER2-positive oesophago-gastric cancer patients treated with trastuzumab lose HER2 positivity (25, 26). Resistance to trastuzumab may also occur through HER2 receptor mutation, such as amino-truncation (27, 28): p95HER2 is an amino-truncated form of the HER2 receptor that lacks the trastuzumab binding site and is found in 60–77% of HER2-positive oesophago-gastric cancer patients (27, 28). Alterations to intracellular signalling pathways, including phosphatidylinositol-4,5-bisphosphate 3-kinase catalytic subunit alpha (PIK3CA)/phosphatase and tensin homolog (PTEN)/PI3K/Ak strain transforming (Akt)/mTOR, may be involved in trastuzumab resistance (29). Epigenetic mechanisms, including micro-ribonucleic acids (microRNAs), can affect these intracellular signalling pathways and may be involved in trastuzumab resistance in oesophago-gastric cancer (30, 31).

Micro-ribonucleic acids (microRNAs, miRs) are small non-coding ribonucleic acids (RNAs) 20–25 nucleotides in length that control gene expression through messenger RNA (mRNA) degradation and post-transcriptional inhibition, influencing cellular pathways, including proliferation, differentiation, apoptosis, and drug sensitivity (32–34). They bind to a ‘seed sequence’, which is a complementary sequence on target mRNA, usually in the 3′ untranslated region (UTR), causing translational repression or mRNA degradation, or upregulating translation if they bind to promoter sequences (34). MiRs are frequently dysregulated in solid and haematological malignancies, including oesophago-gastric cancer (35–40).

MiRs can directly enter the bloodstream to become circulating miRs as a by-product of cell degradation via tumour invasion and angiogenesis (34). Additionally, they can be actively secreted through packaging in lipid vesicles, binding with RNA-binding proteins, and high-density lipoprotein, allowing them to mediate intercellular gene regulation in a manner analogous to hormone signalling. Circulating miRs are potential biomarkers of burden of disease, response to trastuzumab-based therapy, and progression in HER2-positive breast cancer (41, 42). A serum-based four-circulating-miR signature (miR-940, miR-451a, miR-15-5p, and miR-17-3p) is predictive of the response to first-line trastuzumab in metastatic HER2-positive breast cancer patients (43). Another study in HER2-positive early breast cancer reported that early dynamic changes in miR-140-5p are associated with trastuzumab response (44). Circulating miRs can demonstrate conflicting results: for example, circulating-tumour-miR-210 has been associated with trastuzumab response (45) and resistance (46) in HER2-positive breast cancer patients. To our knowledge, our study is the first to evaluate circulating miRs as biomarkers of response to trastuzumab-based therapy in HER2-positive advanced oesophago-gastric cancer patients (47).

This study aimed to identify miRs involved in trastuzumab resistance and evaluate whether circulating miRs might represent a clinically useful biomarker to predict which patients are likely to respond and which patients might display resistance to first-line trastuzumab-based therapy in advanced HER2-positive oesophago-gastric cancer. This might allow personalisation of treatment. To address this question, we used a bench-to-bedside approach, initially performing a high-throughput 1015 Exiqon miRCURY locked nucleic acid (LNA)™ microRNA inhibitor library screen in trastuzumab-treated HER2-positive NCI-N87 and HER2 negative FLO-1 oesophago-gastric cancer cell lines to shortlist candidate microRNAs that affect cell viability when treated with trastuzumab-based therapy. To evaluate which of these shortlisted miRs displayed higher tissue expression in HER2-positive patient tumour samples than in HER2-negative patient tumour samples (suggesting a relevance to the HER2 signalling pathway), we performed NanoString nCounter^®^ miR analysis in NCI-N87, FLO-1, and MAGIC trial (ISRCTN93793971) formalin-fixed paraffin-embedded (FFPE) oesophago-gastric cancer patient samples. Having established a candidate miR, we evaluated circulating miR expression of our shortlisted miR within two clinical trial sub-studies: FOrMAT (NCT02112357) (48) and Arm B1 of the Phase 2 open label multicentre randomised ‘PLAnning Treatment For Oesophago-Gastric Cancer: a Randomised Maintenance Therapy Trial’ (PLATFORM) (NCT02678182) (49). We correlated expression of our candidate miR with the clinical outcomes overall survival (OS), progression-free survival (PFS), and 3-month progression-free rates (PFR) to evaluate whether our shortlisted miR represented a clinically useful biomarker to predict resistance to trastuzumab-based therapy in HER2-positive advanced oesophago-gastric cancer patients.

## Methods

### Cell culture

HER2-positive NCI-N87 and OACP4C oesophago-gastric cancer cell lines and a HER2-negative FLO-1 oesophago-gastric cancer cell line were grown under standard culture conditions. Mycoplasma testing was performed prior to experiments and at 6-month intervals.

### High-throughput LNA™ miR-inhibitor screen

Reverse transfection was performed in white flat-bottomed 384-well plates (Corning, NY, USA) using DharmaFECT2 (Dharmacon Life Sciences) for NCI-N87 (volume 0.05 μl per well) and RNAiMAX (ThermoFisherScientific) for FLO-1 (0.15 μl per well). An Exiqon miRCURY locked nucleic acid (LNA)™ microRNA inhibitor library of 1,015 microRNA inhibitors, short interfering RNAs (siRNAs), and siRNA-mediated polo-like kinase 1 (siPLK1) positive controls (LNA-negative control A, LNA-negative control B, and positive control TOX siRNA [siTOX, Dharmacon Life Sciences]) were added using an Echo 550 Liquid Handler (Labcyte) (volume of 500 nl, concentration 50 nM per well). Plates were centrifuged at 1,000 rpm for 1 min and allowed to rest at room temperature for 30 min; then, 2,000 NCI-N87 cells per well and 600 FLO-1 cells per well were plated (total volume of 45 μl per well), centrifuged at 1,000 rpm for 1 min, and then incubated at 37°C (5% CO_2_) for 48 h. Cells were treated with trastuzumab (Genetech Inc, obtained via Material Transfer Agreement) and cisplatin and 5FU chemotherapy (Sigma-Aldrich) based on previous half-maximal inhibitory concentration (IC50) experiments (NCI-1.136 μM N87 cisplatin, 1.02 μM 5FU, 1.33 μg/ml trastuzumab, 2.94 μM FLO-1 cisplatin, 1.4 μM 5FU, 1.56 μg/ml trastuzumab). Plates were centrifuged at 1,000 rpm for 1 min and then incubated at 37°C (5% CO_2_) for 72 h. Cell viability was assessed using a homogeneous fluorescent assay: 5 μl/well Cell Titer Blue (Promega, Madison, WI, USA) was added to each well and plates were briefly centrifuged at 1,000 rpm for 1 min and then incubated at 37°C (5% CO_2_) for 3 h; then, fluorescence was measured using the Envision spectrophotometer (PerkinElmer, Waltham, MA, USA). Three replicates were performed for trastuzumab plus chemotherapy, and a single-replicate for single-agent trastuzumab.

### Large-scale screen analysis methods

Microsoft Excel was used to analyse the cell viability results. Results were averaged across all five plates and across all three replicates for each cell line and normalised to LNA-negative control B. MiRs were considered significant if inhibition with LNAs resulted in a more than 40% reduction in cell viability (CV <0.6) relative to controls at 72 h following treatment, and a t-test *p*-value of <0.001 was obtained across three biological replicates in each cell line.

### Total RNA extraction from formalin-fixed paraffin-embedded patient tumour samples

MAGIC tumour samples (ISRCTN93793971) were macrodissected to enrich for tumour content. Total RNA extraction was performed using a RecoverAll™ Total Nucleic Acid Isolation Kit for FFPE (Thermo Fisher Scientific, Waltham, MA, USA) according to the manufacturer’s protocol.

### RNA extraction from plasma and serum

For the FOrMAT trial, RNA was extracted from 300 μl of serum and plasma samples using an Exiqon miRCURY RNA Isolation Kit-Biofluids (Exiqon, Vedbaek, Denmark) according to the manufacturer’s protocol. For the PLATFORM trial, RNA was extracted from plasma using a Qiagen miRNeasy Serum/Plasma Advanced Kit (Cat# 217204, Qiagen, Hilden, Germany) as per the manufacturer’s protocol.

### NanoString nCounter^®^ analysis

NanoString nCounter^®^ analysis was performed with Human miRNA Panel v2 (Category number GXA-MIR2-12) in extracted RNA from NCI-N87 and FLO-1 oesophago-gastric cancer cell lines, and in extracted RNA from HER2-positive and HER2-negative MAGIC trial (ISRCTN93793971) patient FFPE samples. Samples were prepared and hybridised as per the NanoString nCounter^®^ miRNA Expression Assay User Manual. A numerical value of 50 or greater represents high expression.

### Digital droplet polymerase chain reaction

A standard volume of 5 μl of eluted RNA template for each sample was used for retrotranscription with the specific reverse transfection (RT) probe for miR-148-3p (4366597, Thermo Fisher Scientific) and control miR-16-5p (A25576, Thermo Fisher Scientific) with a TaqMan™ MicroRNA reverse transcription kit (Life Technologies, Thermo Fisher Scientific) using the standard manufacturer’s protocol. RT template (5 μl) was used for digital droplet polymerase chain reaction (ddPCR) with polymerase chain reaction (PCR) probe TaqMan™ (Assay ID, 000470; miRBase accession number MI0000253; Thermo Fisher Scientific) and Bio-Rad mastermix for probes (Bio-Rad, USA). Absolute quantification of miR-148a-3p and miR-16-5p was performed using a ddPCR standard Bio-Rad protocol for droplet generation and a thermal protocol for probes (Bio-Rad, USA). Absolute miR-148a-3p values were divided by absolute miR-16-5p values to create normalised miR-148a-3p (NmiR-148a-3p).

### Evaluation in trial patients, data collection, and endpoints

The FOrMAT trial (NCT02112357) is a single-institution feasibility study enrolling patients with advanced gastrointestinal (GI) malignancies at the Royal Marsden Hospital (48). The primary endpoint was the percentage of patients in whom a currently actionable molecular alteration was detected by genetic sequencing.

The PLATFORM trial is a phase 2 open label multicentre randomised trial (NCT02678182). The primary objective of the PLATFORM trial was to evaluate the effects on PFS of maintenance therapies following the completion of standard first-line chemotherapy in patients with HER2-positive and HER2-negative locally advanced or metastatic oesophago-gastric adenocarcinomas. Arm B1 of the PLATFORM trial recruited patients with HER2-positive locally advanced or metastatic oesophago-gastric adenocarcinomas and these patients were treated with standard-of-care therapy (first-line trastuzumab-based chemotherapy followed by maintenance trastuzumab).

Informed consent was obtained for all patients. Clinical data collected included sex, age, location of primary tumour, location of initial metastases (if applicable), HER2 amplification status, chemotherapy backbone, site(s) of progression of disease (PD), PFS, OS, duration of trastuzumab therapy, and response at 3 months from initiation of therapy using RECIST 1.1 criteria from baseline.

Our hypothesis for FOrMAT and PLATFORM sub-studies was that HER2-positive advanced oesophago-gastric cancer patients with HIGH (>median) baseline circulating plasma miR-148a-3p levels may experience shorter OS and PFS.

Within the FOrMAT and PLATFORM sub-studies, OS as primary objective and PFS as secondary objective assessed high baseline plasma miR-148a-3p levels versus low miR-148a-3p levels in advanced HER2-positive oesophago-gastric cancer patients treated with trastuzumab. OS as a primary endpoint was calculated from cycle 1 day 1 systemic therapy until the date of death from any cause. Patients alive at the time of analysis were censored at the date of last follow-up. The secondary endpoint, PFS, was calculated from the date of cycle 1 day 1 systemic therapy until the date of disease progression according to imaging (response evaluation criteria in solid tumors [RECIST] 1.1 criteria), clinical progression, or death from any cause, whichever occurred first. Progression events were determined by local investigator assessment. Patients lost to follow-up or who remained alive without disease progression at the time of analysis were censored at the date of last follow-up.

A further secondary objective within the FOrMAT sub-study was to explore whether patients categorised as ‘responders’ had lower circulating miR-148a-3p levels than those defined as ‘non-responders’. Non-responders were defined as patients who experienced disease progression using RECIST 1.1 imaging assessment after 6 or 8 cycles of trastuzumab-based therapy. Responders were defined as patients who experienced partial response or stable disease using RECIST 1.1 after 6 or 8 cycles of trastuzumab-based therapy.

An additional secondary objective within the PLATFORM sub-study was to assess whether patients with high baseline plasma miR-148a-3p levels experienced significantly poorer progression-free rates (PFRs) at 3 months than those with low baseline plasma miR-148a-3p levels. The secondary endpoint, PFR, at 3 months post-randomisation was evaluated according to RECIST 1.1 criteria as the proportion of patients with a complete response (CR), partial response (PR), or stable disease (SD). For patients with no measurable disease, absence of clinical or radiological progression was classed as stable disease. The response was determined by local investigator assessment. Patients with no available scan were excluded from the PFR analysis unless they were known to have clinically progressed, stopped treatment due to progression, or died prior to the scan (included as having disease progression).

### Evaluation in trial patients: statistical analysis

Within FOrMAT and PLATFORM sub-studies, all HER2-positive patients who underwent registration and/or randomisation and were treated with first-line trastuzumab-based therapy and with analysable samples and available clinical information were included in the analysis.

Demographic and clinical characteristics were summarised both overall and by exposure group (LOW versus HIGH miR-148a-3p). Categorical variables were described using frequencies and percentages. Continuous variables were described using mean and standard deviation if symmetrically distributed, or median and interquartile range (IQR) if the data were skewed.

Within biomarker subgroups (miR-148a-3p HIGH versus miR-148a-3p LOW), survival curves were generated using the methods of Kaplan and Meier. The log-rank test was used to compare the survival functions of miR-148a-3p HIGH and miR-148a-3p LOW patient subgroups. Median survival and survival estimates at 6, 12, and 24 months are presented with a 95% confidence interval (CI). A hazard ratio (HR) along with its 95% CI and p value are reported to compare patient subgroups of miR-148a-3p HIGH and miR-148a-3p LOW, calculated from Cox regression models.

Multivariable cox regression was performed when miR-148a-3p HIGH versus miR-148a-3p LOW was significant univariately, including but not limited to primary tumour location (oesophagus/oesophago-gastric junction/stomach), extent of disease (locally advanced/metastatic), sites and number of metastases, Eastern Cooperative Oncology Group (ECOG) performance status, and the systemic therapy regimen used. MiR-148a-3p data were compared between ‘responder’ and ‘non-responder’ subgroups by calculating the mean values of each group using t-tests.

A sensitivity analysis evaluated whether normalisation to miR-16-5p (where available) represented an effective technique. Another sensitivity analysis evaluated whether early dynamic changes in miR-148a3p or NmiR-148a-3p were associated with OS, PFS, or PFR. Statistical analysis was performed using Microsoft Excel, GraphPad Prism 7, and Stata 17.

## Results

### Large-scale MicroRNA inhibitor screen results

**Figure 1:**
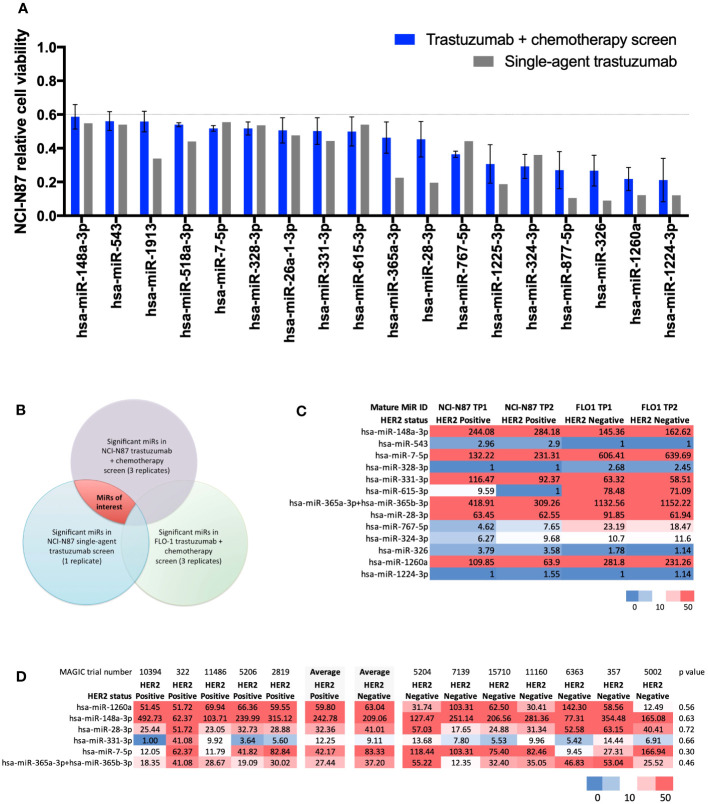
Results from a large-scale microRNA inhibitor screen and NanoString nCounter^®^ analysis. **(A)** Statistically significant miRs relevant to the HER2-signalling pathway identified in a high-throughput large-scale RNA interference screen. Bar chart showing 18 microRNAs (miRs) causing a statistically significant (t-test p<0.001) decrease in relative cell viability (CV<0.6) relative to controls in the HER2-positive NCI-N87 cell line when treated with trastuzumab plus chemotherapy (n=3) and single-agent trastuzumab (n=1). In order of greatest to least effect on cell viability when inhibited: miR-1224-3p, miR-1260a, miR-326, miR-877-5p, miR-324-3p, miR-1225-3p, miR-767-5p, miR-28-3p, miR-365a-3p, miR-615-3p, miR-331-3p, miR-26a-1-3p, miR-328-3p, miR-7-5p, miR-518a-3p, miR-1913, miR-543, and miR-148a-3p. **(B)** Venn diagram showing the methodology for identifying significant miRs in the trastuzumab-signalling pathway. MiRs of interest are indicated by the area shaded in red. **(C)** NanoString nCounter^®^ analysis with Human miRNA Panel v2 in HER2-positive NCI-N87 and HER2-negative FLO-1 cell lines for shortlisted miRs. Heatmap of normalised microRNA expression at timepoint 1 (TP1) and timepoint 2 (TP2) in the HER2-positive oesophago-gastric cell line NCI-N87 and HER2-negative oesophago-gastric cell line FLO-1. TP2 was two passages after TP1. Six miRs showed high expression (>50) in the NCI-N87 cell line: miR-148a-3p, miR-7-5p, miR-331-3p, miR-365a-3p, miR-28-3p, and miR-1260a. MiR-148-3p and miR-331-3p were more highly expressed in the HER2-positive NCI-N87 cell line than in the HER2-negative FLO-1 cell line. **(D)** NanoString nCounter^®^ analysis of MAGIC trial FFPE samples. Heatmap showing NanoString nCounter normalised miR expression results for miR-148a-3p, miR-7-5p, miR-331-3p, miR-365a-3p, miR-28-3p, and miR-1260a in HER2-positive (n=5) and -negative (n=7) MAGIC trial patients. MiR-148a-3p and miR-331-3p had a numerically higher average expression level in HER2-positive patients than in HER2-negative patients.

Results from the high-throughput large-scale RNA interference screen using a library of LNA™ miR-inhibitors identified 18 statistically significant miRs relevant to the HER2-signalling pathway (**Figure 1A**). In order of greatest to least effect on cell viability when inhibited, these 18 miRs were: miR-1224-3p, miR-1260a, miR-326, miR-877-5p, miR-324-3p, miR-1225-3p, miR-767-5p, miR-28-3p, miR-365a-3p, miR-615-3p, miR-331-3p, miR-26a-1-3p, miR-328-3p, miR-7-5p, miR-518a-3p, miR-1913, miR-543, and miR-148a-3p. These microRNAs, when inhibited, resulted in a fall in cell viability compared with controls. A Venn diagram (**Figure 1B**) outlines the criteria for shortlisting. Full screen results are shown in **Supplementary Figure 1**.

### NanoString nCounter^®^ analysis

NanoString nCounter^®^ analysis with Human miRNA Panel v2 in HER2-positive NCI-N87 and HER2-negative FLO-1 cell lines for shortlisted miRs (**Figure 1A**) found six miRs with high expression (>50) in the NCI-N87 cell line: miR-148a-3p, miR-7-5p, miR-331-3p, miR-365a-3p, miR-28-3p, and miR-1260a (**Figure 1C**). Of these, miR-148-3p and miR-331-3p demonstrated higher expression in the HER2-positive NCI-N87 cell than in the HER2-negative FLO-1 cell line, suggesting a relevance to HER2 biology.

**Figure 2:**
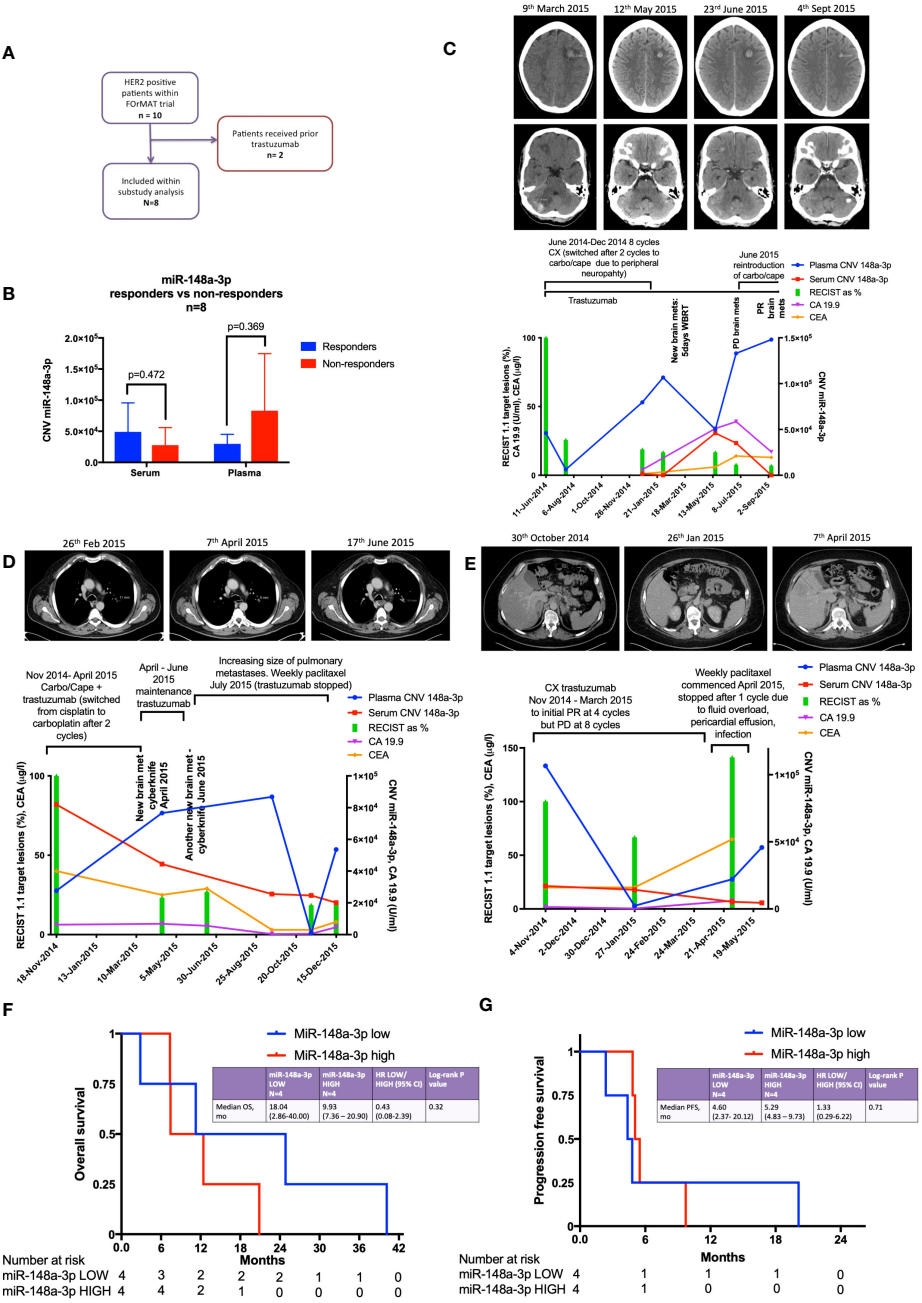
Results from the FOrMAT trial sub-study for advanced HER2-positive oesophago-gastric cancer patients. **(A)** FOrMAT trial CONSORT diagram. **(B)** Baseline CNV for serum and plasma miR-148a-3p in ‘responders’ versus ‘non-responders’ in the FOrMAT trial (n=8). The solid bars represent mean ‘responder’ CNV and ‘non-responder’ CNV. The error bars show the maximum miR-148a-3p CNV in ‘responders’ and ‘non-responders’. **(C)** FOrMAT trial patient 020. Graph showing plasma (blue line) and serum (red line) miR-148-3p CNV correlated with RECIST 1.1 target lesions (green bars), CA 19.9 (purple line), and CEA (orange line). The appearance of new brain metastases (left frontal and right cerebellar) is noted on the graph and shown in the CT images above: these new brain metastases are not measured as part of the original target lesions but signify disease progression. **(D)** FOrMAT trial patient 092. Graph showing plasma (blue line) and serum (red line) miR-148-3p CNV correlated with RECIST 1.1 target lesions (green bars), CA 19.9 (purple line), and CEA (orange line). CT images demonstrating the change in size of a target lung lesion are shown. This patient had brain metastases that were treated with CyberKnife. These new brain metastases were not measured as part of the original RECIST 1.1 target lesions but signify disease progression. **(E)** FOrMAT trial patient 085. Graph showing plasma (blue line) and serum (red line) miR-148-3p CNV correlated with RECIST 1.1 target lesions (green bars), CA 19.9 (purple line), and CEA (orange line). CT imaging of a target lesion in the liver is shown. **(F)** Kaplan–Meier overall survival (OS) curves for plasma MiR-148a-3p low versus MiR-148a-3p high patients within the FOrMAT trial (n=8). Median OS for the miR-148a-3p ‘low’ subgroup was 549 days (18.04 months) versus 302 days (9.93 months) (p=0.322) for the miR-148a-3p ‘high’ subgroup (HR, 0.43; 95% CI, 0.08–2.39). **(G)** Kaplan–Meier progression-free survival (PFS) curves for plasma MiR-148a-3p low versus high patients within the FOrMAT trial (n=8). Median PFS for the miR-148a-3p ‘low’ subgroup was 140 days (4.60 months) versus 161 days (5.29 months) for the miR-148a-3p ‘high’ subgroup (p=0.714; HR, 1.33; 95% CI 0.29–6.22).

NanoString nCounter^®^ analysis of MAGIC trial FFPE samples from HER2-positive (n=5) and HER2-negative (n=7) patients found that miR-148a-3p and miR-331-3p had a numerically higher average expression level in HER2-positive patients than in HER2-negative patients (**Figure 1D**). Validation experiments in the HER2-positive NCI-N87 cell line demonstrated that the inhibition of miR-148a-3p caused a greater difference (delta, Δ) in cell viability than the inhibition of any of the other shortlisted miRs (miR-7-5p, miR-331-3p, miR-365a-3p, and miR-28-3p) compared with controls in the HER2-positive oesophago-gastric cell line NCI-N87 when treated with trastuzumab and chemotherapy (Δ 67%, p=0.17, **Supplementary Figure 2A**) or single-agent trastuzumab (Δ46%, p=0.39, **Supplementary Figure 2B**). A further validation experiment in the HER2-positive OACP4C cell line demonstrated that miR-148a-3p inhibition resulted in Δ50% when treated with trastuzumab and chemotherapy (**Supplementary Figure 2C**) and Δ11% with single-agent trastuzumab (**Supplementary Figure 2D**). These findings led to miR-148a-3p being selected for further experiments. RNA sequencing did not identify any pathway effect of miR-148a-3p inhibition (see **Supplementary Figure 2**). Some genes were upregulated or downregulated when miR-148a-3p was inhibited (see **Supplementary results Figure 2**), but these genes were not consistent across the two HER2-positive oesophago-gastric cancer cell lines explored in our study.

### Translational sub-study results

#### FOrMAT trial

There were ten HER2-positive oesophago-gastric FOrMAT trial patients (a total of 35 clinical timepoints) with serum and plasma samples available for analysis. Two patients had received trastuzumab previously; therefore, they were excluded from subsequent sub-study analysis, as shown in the flow diagram in **Figure 2A**. The MiR-148a-3p (n=8) copy number variation (CNV) (copies per 20 μl) mean was 59,971 (median, 36,810; IQR, 12,455–87,550; range, 152–224,000). The CNV copies per μl mean was 3,153.2 (median, 1,840.50; IQR, 622.75–4,377.5; range, 7.6–11,200).

Baseline patient characteristics are shown in **Table 1**. MiR-148a-3p LOW was defined as CNV<36,810 copies per 20 μl (<1,840.50 copies per 1 μl) (n=4) and miR-148-3p HIGH was defined as ≥36,810 per 20 μl (≥1,840.50 copies per 1 μl) (n=4).

**Table 1:** FOrMAT trial patient characteristics.

	miR-148a-3p LOW N=4		miR-148a-3p HIGH N=4		Overall N=8	
	N	%	N	%	N	%
Age						
Mean (SD)	49.8	11.5	49.3	12.0	49.5	10.9
Median (IQR)	50.5	(42.5 to 57)	47.0	(40.0 to 58.5)	50.5	(40.0 to 57.5)
Range		(35.0 to 63.0)		(38.0 to 65.0)		(35.0 to 65.0)
Gender						
Female	0	0	0	0	0	0
Male	4	100	4	100	8	100
Primary Site						
OG Junction	2	50	1	25	3	37.5
Oesophagus	1	25	3	75	4	50
Stomach	1	25	0	0	1	12.5
Extent of disease						
Locally advanced	1	25	1	25	2	25
Metastatic	3	75	3	75	6	75
**Number of metastatic sites** ≤1	2	50	1	25	3	37.5
≥2	2	50	3	75	5	62.5
Sites of metastatic disease (y/n) for n=8						
Liver	1	25	3	75	4	50
Peritoneum	1	25	0	0	1	12.5
Distant nodes	1	25	2	50	3	37.5
Bone	1	25	1	25	2	25
Lung	0	0	1	25	1	12.5
Adrenal	0	0	1	25	1	12.5
Number of trastuzumab cycles received during induction chemo						
3	1	25	0	0	1	12.5
6	1	25	1	25	2	25
7	0	0	1	25	1	12.5
8	2	50	2	50	4	50
Systemic therapy regimen used						
Capecitabine/cisplatin/trastuzumab	4	100	4	100	8	100

There was no statistically significant relationship between responders versus non-responders for miR-148a-3p LOW versus HIGH (**Figure 2B**). FOrMAT trial non-responder patients demonstrated mean plasma CNV miR-148-3p of 83,090 compared with mean plasma CNV miR-148-3p of 29,687 in responders (**Figure 2B**) (p=0.369). Mean serum miR-148a-3p CNV in non-responders was 27,784 and in responders mean miR-148a-3p CNV was 49,040 (p=0.472).

FOrMAT trial patient 020 (**Figure 2C**) was initially treated with a chemotherapy backbone of cisplatin and capecitabine (CX) but was switched to carboplatin in combination with capecitabine after two cycles due to peripheral neuropathy. The patient demonstrated an excellent response at the initial computerised tomography (CT) assessment with a 74% reduction in target lesions. MiR-148a-3p plasma CNV decreased in line with this response but subsequently began to steadily increase, reaching 106,600 in February 2015, at which point the patient was found to have brain metastases. After radiotherapy to these brain metastases, plasma miR-148a-3p levels decreased but then increased again at the point that brain progression was detected. Plasma miR-148a levels continued to increase and chemotherapy was reintroduced. Trastuzumab continued throughout. Systemic disease remained well controlled throughout this time. Serum miR-148a-3p CNV, carbohydrate antigen 19.9 (CA 19.9), and carcinoembryonic antigen (CEA) were not available for all timepoints but serum miR-148a-3p CNV and CA 19.9 began to increase at the time of brain progression then decreased as systemic chemotherapy was initiated, and CEA increased at the time of brain progression.

FOrMAT trial patient 085 (**Figure 2D**) commenced CX and trastuzumab to initial PR at four cycles but PD at eight cycles. Plasma miR-148a-3p levels decreased as disease burden reduced and increased when systemic disease progressed. Serum miR-148a-3p levels decreased as disease progression was identified by CT. CA 19.9 and CEA began to increase from January onwards but blood samples were not available for all timepoints. CT images showed a liver lesion that decreased in size in January 2015 after four cycles of systemic therapy but grew in size in April 2015 after eight cycles.

FOrMAT trial patient 092 (**Figure 2E**) initially received two cycles of CX then switched to carboplatin and capecitabine alongside trastuzumab. The patient demonstrated a good systemic response on CT and entered a period of maintenance trastuzumab. However, the patient developed brain metastases, which were treated with CyberKnife in April and June 2015. CT imaging in June 2015 showed that the lung lesions had increased in size; therefore, trastuzumab was stopped and weekly paclitaxel commenced. Serum miR-148a-3p levels decreased in line with systemic CT findings demonstrating a response. Plasma miR-148a-3p levels increased at the time of brain progression, decreased after weekly paclitaxel was introduced, and then began to increase again. CA 19.9 decreased during first-line carboplatin and capecitabine and trastuzumab despite the new brain metastasis, increased during maintenance trastuzumab, decreased during paclitaxel chemotherapy, and began to increase again towards the end of treatment. CEA decreased slightly during second-line paclitaxel treatment and then increased at the end of treatment.

There was no statistically significant relationship between OS or PFS for miR-148a-3p LOW versus HIGH (**Figures 2F, G**). Kaplan–Meier survival curves for OS (**Figure 2F**) and PFS (**Figure 2G**) (n=8) according to miR-148a-3p low versus miR-148a-3p high demonstrate that the median OS for the miR-148a-3p ‘low’ subgroup was 549 days (18.04 months) versus 302 days (9.93 months) (p=0.322) for the miR-148a-3p ‘high’ subgroup (HR, 0.43; 95% CI, 0.08 to 2.39). Median PFS for the miR-148a-3p ‘low’ subgroup was 140 days (4.60 months) versus 161 days (5.29 months) for the miR-148a-3p ‘high’ subgroup (p=0.714; HR, 1.33; 95% CI, 0.29 to 6.22).

#### PLATFORM trial

**Figure 3:**
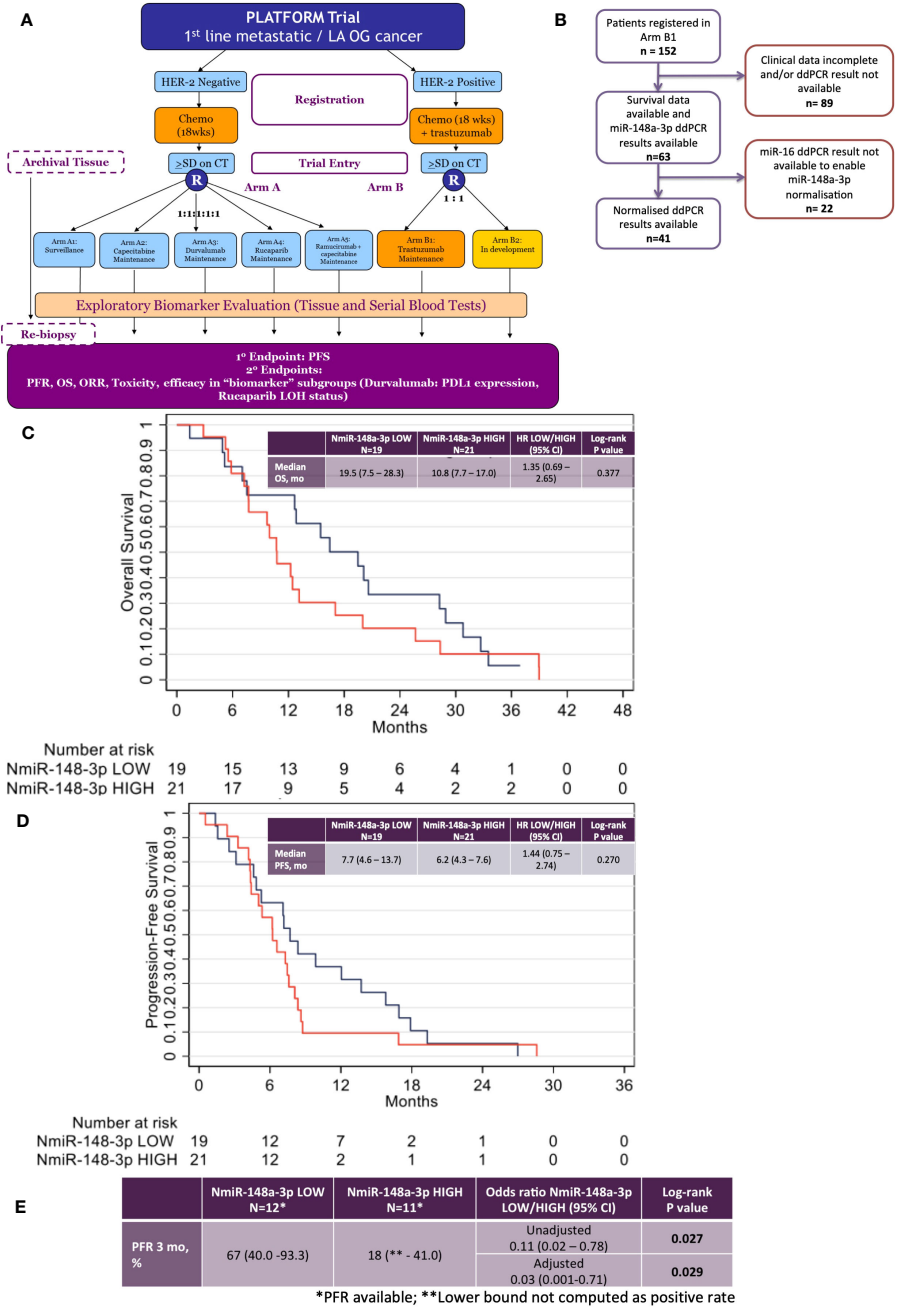
Results from the phase 2 PLATFORM trial sub-study for advanced HER2-positive oesophago-gastric cancer patients. **(A)** PLATFORM trial CONSORT diagram. **(B)** MiR-148a-3p and normalised miR-148a-3p (NmiR-148a-3p) sub-study CONSORT within the PLATFORM trial. **(C)** Kaplan–Meier curve of overall survival (OS) normalised miR-148a-3p (NmiR-148a-3p) LOW versus HIGH. **(D)** Kaplan–Meier curve of progression-free survival (PFS) NmiR-148a-3p LOW versus HIGH. **(E)** Progression-free rates (PFR) for NmiR-148a-3p LOW versus HIGH.

The PLATFORM trial schema is shown in **Figure 3A** and the sub-study schema is shown in **Figure 3B**. A total of 63 patients were eligible for inclusion in the sub-study analysis. Median follow-up was 38 months with a data cutoff of August 2022.

The data had a strong positive skew, although the range was above zero: miR-148a-3p (n=63) mean was 1,302.7 CNV (miR copies per microlitre) (median, 1,120; IQR, 227–1,761; range 0.44–7,600). MiR-148a-3p LOW was defined as CNV <1,120 (n=31) and miR-148-3p HIGH was defined as ≥1,120 (n=32).

A total of 41 patient results could be normalised to miR-16-5p; the remainder did not have miR-16-5p values available. Normalised miR-148a-3p (NmiR-148a-3p) was calculated by dividing miR-148a-3p CNV by miR-16-5p CNV. The NmiR-148-3p (n=41) mean was 0.192, (median, 0.175; IQR, 0.070–0.245; range 0.001–0.759). A sensitivity analysis was performed on all endpoints using a binary cutoff of the median normalised miR-148a-3p (median NmiR-148a-3p = 0.175), NmiR-148a-3p LOW (<0.175, n=20), and NmiR-148-3p HIGH (≥0.175, n=21). Baseline patient characteristics are shown in **Table 2**.

**Table 2:** PLATFORM sub-study HER2-positive patient characteristics.

	miR-148a-3p LOW N=31		miR-148a-3p HIGH N=32		Overall N=63	
	N	%	N	%	N	
Age						
Mean (SD)	62.0	13.8	63.0	12.3	62.0	13.0
Median (IQR)	68.0	(54.0 to 70.0)	66.0	(56.0 to 71.0)	67.0	(55.0 to 70.0)
Range		(30.0 to 82.0)		(36.0 to 82.0)		(30.0 to 82.0)
Gender						
Female	1	3	4	13	5	8
Male	30	97	28	88	58	92
Primary Site						
OG Junction	11	35	11	34	22	35
Oesophagus	15	48	16	50	31	49
Stomach	5	16	5	16	10	16
Extent of disease						
Locally advanced	2	6	2	6	4	6
Metastatic	29	94	30	94	59	94
**Number of metastatic sites** ≤1	24	77	27	84	51	81
≥2	7	23	5	16	12	19
Sites of metastatic disease (y/n) for n=59						
Liver	18	62	15	50	33	56
Peritoneum	3	10	2	7	5	8
Distant nodes	10	34	13	43	23	39
Bone	1	3	3	10	4	7
Lung	9	31	10	33	19	32
Omentum	0	0	1	3	1	2
Other*	0	0	3	10	3	5
Number of trastuzumab cycles received during induction chemo						
0	0	0	1	3	1	2
2	0	0	1	3	1	2
3	0	0	2	6	2	3
4	4	13	2	6	6	10
5	2	6	2	6	4	6
6	22	71	22	69	44	70
7	1	3	1	3	2	3
8	2	6	1	3	3	5
Systemic therapy regimen used						
5-FU/cisplatin/trastuzumab	1	3	2	6	3	5
Capecitabine/cisplatin/trastuzumab	21	72	24	75	45	74
Other	7	24	6	19	13	21

*Other metastatic sites listed are abnormal soft tissue anterior to the abdominal aorta, supraclavicular node, and tracheal nodes.

There was no statistically significant relationship between OS or PFS for NmiR-148a-3p (**Figures 3C, D**) or miR-148a-3p LOW versus HIGH (**Supplementary Figures 3A, B**). Among 40 patients evaluable for NmiR-148a-3p, median OS in the NmiR-148a-3p low group was 19.5 months (95% CI: 7.5-28.3) compared with 10.8 months (95% CI: 7.7–17.0) in the NmiR-148a-3p high group (HR, 1.35 (95% CI, 0.69–2.65; n=40; p=0.377) (**Figure 3C**). Median PFS in the NmiR-148a-3p low group was 7.7 months (95% CI: 4.6-13.7) compared with 6.2 months (95% CI, 4.3–7.6) in the NmiR-148a-3p high group (HR, 1.44 (95% CI, 0.75–2.74); n=40; p=0.270 (**Figure 3D**).

Normalised miR-148a-3p (NmiR-148a-3p) LOW versus HIGH demonstrated a statistically significant difference in PFR at 3 months: 67% (95% CI, 40.0–93.3) in the NmiR-148a-3p low group compared with 18% (95% CI, lower bound not computed as a positive rate, −41.0) in the NmiR-148a-3p high group (OR, 0.11 (0.02–0.78); n=23; p=0.027). A model adjusting for primary tumour site, metastatic disease, and number of sites demonstrated a statistically significant difference in PFR at 3 months (aOR, 0.03; 95% CI, 0.001–0.71; p=0.029) (**Figure 3E**).

Median OS in the miR-148a-3p low group was 12.8 months (95% CI, 7.8–17.0) compared with 12.4 months (95% CI, 9.0–20.0) in the miR-148a-3p high group (HR, 0.98 (95% CI, 0.57–1.66); n=62; p=0.933) (**Supplementary Figure 3A**). Median PFS in the miR-148a-3p low group was 6.7 months (95% CI, 4.6–8.4) compared with 7.2 months (95% CI, 4.3–7.9) in the miR-148a-3p high group (HR, 1.08 (95% CI, 0.65–1.81); n=62; p=0.759 (**Supplementary Figure 3B**). The PFR at 3 months was 53% (95% CI, 29.2–76.7) in the miR-148a-3p low group compared with 43% (95% CI, 16.9–68.8) in the miR-148a-3p high group (OR, 0.67 (95% CI, 0.2–2.8); n=31; p=0.577) (**Supplementary Figure 3C**).

### Exploratory analysis: dynamic changes in circulating miR-148a-3p levels

An additional analysis explored the effect of early dynamic changes in circulating miR-148a-3p levels. Patients were divided into two groups: those with a reduction in miR-148a-3p between registration and C1/C2 (only using C1 if C2 was not available) (N=14), and those with an increase in miR-148a-3p between registration and C1/C2 (only using C1 if C2 was not available) (N=15). There was no statistically significant difference in OS, PFS, or PFRs between these two groups. A limitation is the small numbers. A further analysis was performed in which patients were divided into two groups according to NmiR-148a-3p: those with a reduction in NmiR-148a-3p between registration and C1/C2 (only using C1 if C2 was not available) (N=15), and those with an increase in NmiR-148a-3p between registration and C1/C2 (only using C1 if C2 was not available) (N=6). Numbers between these two groups were less balanced; however, again, there was no statistically significant difference in OS, PFS, and PFRs between these two groups. Additionally, it was noticeable that some patients who experienced an increase in raw miR-148a-3p values experienced a decrease in NmiR-148a-3p, highlighting the importance of effective normalisation techniques.

## Discussion

This is the first study exploring miRs associated with resistance to trastuzumab-based chemotherapy in HER2-positive oesophago-gastric cancer cell lines using large-scale screening methods followed by an evaluation of circulating miR-148a-3p in HER2-positive advanced oesophago-gastric cancer patients treated with trastuzumab-based chemotherapy. We did not identify any significant effect of NmiR-148a-3p or miR-148a-3p HIGH versus LOW on PFS or OS in FOrMAT or PLATFORM clinical trial sub-studies.

Our preplanned sensitivity analysis of NmiR-148a-3p in the PLATFORM sub-study identified a statistically significant difference in 3-month PFRs between normalised miR-148a-3p LOW versus HIGH in HER2-positive oesophago-gastric cancer patients treated with maintenance trastuzumab after the completion of trastuzumab-based chemotherapy. Patients with HIGH NmiR-148a-3p have 0.03 times the odds of being progression-free at 3 months from the commencement of maintenance trastuzumab after the completion of trastuzumab-based chemotherapy than patients with LOW NmiR-148a-3p, when adjusted for primary tumour site, metastatic disease, and number of sites.

MiR-148a-3p has a 68 nucleotide sequence located on chromosome 7p15.2 and a stem-loop structure sequence located on the negative strand of chromosome 7:25949919-25949986 (50, 51). MiR-148a-3p is frequently described as exerting a tumour suppressor effect (38, 52, 53). Studies in HER2-positive early breast cancer demonstrate that early dynamic changes in miR-148a-3p are significantly correlated with pathological complete response rates to trastuzumab-based chemotherapy, with patients whose miR-148a-3p levels increase correlating with higher pathological complete response rates (p=0.008) (42). However, current available evidence for the role of miR-148a-3p in oesophago-gastric cancer yields contradictory results. One study suggests low levels of miR-148a-3p are associated with a shorter disease-free survival (DFS) in gastric cancer patients (54). By contrast, high miR-148a-3p levels are associated with significantly shorter OS and PFS in oesophageal adenocarcinoma patients (55), and miR-148a-3p expression was almost exactly identical in adenocarcinoma histopathological samples to adjacent normal mucosa (55). Target prediction and function of miR-148a-3p reveal a list of 1,637 predicted interactions using miRWalk (56). Ontology/pathway analysis demonstrates that miR-148a-3p can affect PI3k/Akt and MAPK signalling, which are involved in the HER2 signalling pathway (42). Erb-B2 receptor tyrosine kinase 3 (*ERBB3*) (57–59) and its downstream signalling pathways are validated targets of miR-148a-3p (60). Additionally, MiR-148a-3p can affect drug metabolism (42). Ours is the first study to explore circulating miR-148a-3p in HER2-positive advanced oesophago-gastric cancer patients treated with trastuzumab-based chemotherapy. Unfortunately, in our data, we were unable to pick up significant enrichments of any of these pathways. Perhaps a larger panel of cell lines need to be explored to identify pathways that this miR is involved in. This could be explored in a subsequent study.

Our RNA sequencing results did not detect any pathway effect of miR-148a-3p inhibition on the HER2 signalling pathway. We could detect differentially expressed genes and transcripts in the NCI-N87 and OACP4C cell lines but a limitation of our study is that the upregulated and downregulated genes appear to differ between the NCI-N87 and OACP4C cell lines; therefore, the mechanism of action of miR-148a-3p inhibition is not explained. Of interest, miR148a-3p inhibition led to the upregulation of growth arrest DNA-damage inducible 45 alpha (GADD45A) in the OACP4C cell line and upregulation of growth arrest DNA-damage inducible 45 beta (GADD45B) in the NCI-N87 cell line, as compared with controls. GADD45 is a cell cycle regulator and its signalling involves MAPK, which is also involved in HER2 signalling (61, 62). These proteins are involved in responses to cell injury and contribute to p53 activation via p38 (63). Patients with lower levels of miR-148a-3p might have higher GADD45 levels and therefore this might exert an antiproliferative and pro-apoptotic effect on tumour cells, which may affect the response to trastuzumab. Our RNA sequencing results also demonstrated the upregulation of ATPase Na+/K+ transporting subunit beta 3 (ATP1B3) in the NCI-N87 cell line. ATP1B3 is known to be involved in the PI3K/Akt signalling pathway and is therefore relevant to HER2 signalling (64). Further studies to silence and re-express our shortlisted miRs of interest and analyse panels of miRs (42) in additional HER2-positive cell lines might provide valuable information on their role in resistance to trastuzumab-based therapy in HER2-positive oesophago-gastric cancer patients, but this was beyond the scope of this study.

A limitation to our study was the small sample size (n=8 for FOrMAT and n=63 for PLATFORM), which limited the statistical power of our study and affect the p value, limiting the conclusions that can be drawn from this small study. One contributing reason for the small sample size is that only 20% of advanced oesophago-gastric cancers are HER2-positive (4, 5). Another reason is that Arm B1 of the PLATFORM trial is currently a ‘standard-of-care’ arm, which may have affected recruitment. Another limitation to our study was the normalisation of circulating miRs: not all PLATFORM samples could be normalised to miR-16, which further reduced the numbers that could be included in the statistical analysis for NmiR-148a-3p. This might affect the p value of our study and limits the conclusions that can be drawn from this analysis. A further limitation of our study was our normalisation technique. MiR expression levels vary between patients. The FOrMAT data is not normalised to a particular housekeeping miR and therefore the results contain absolute values rather than normalised values. In the PLATFORM study, miR-148a-3p was normalised to miR-16 to calculate NmiR-148a-3p, but as yet, there is no standardised approach for normalising circulating miRs (65). The method by which circulating miRs are collected, processed, and normalised needs to be standardised (34, 66). Normalisation to spiked-in *C. elegans* or the housekeeping controls miR-16, miR-223, or miR-24-3p has been proposed (66, 67). It is unclear whether normalisation to miR-16 represents the best technique for clinical utility (65) and further research studies into the standardisation of normalisation techniques for circulating microRNAs are required.

An interesting finding from the FOrMAT trial analysis was that serum and plasma miR-148a-3p yielded different results for responders versus non-responders. Additionally, plasma samples were available for more clinical timepoints than serum samples, potentially due to the methods of sample collection. Plasma samples were used for the PLATFORM trial due to availability (no serum samples were available for analysis). Differences exist between plasma and serum circulating miRs (68): in a comparison of serum analysis versus plasma analysis, some miRs can be detected in the serum, some in the plasma, and some in both (69–71). Some researchers suggest that plasma and serum are directly comparable for microRNA analysis (71). In the case of circulating miR-148a-3p, there is evidence from studies in breast cancer to suggest that plasma is a suitable choice for measuring circulating miR-148a-3p (42). A systematic review in gastrointestinal cancer supports plasma as a valid choice for measuring circulating miRs (72). The variability between different studies (70, 73) regarding the use of plasma or serum represents a limitation in the use of circulating miRs as biomarkers: the standardisation of techniques must be achieved to allow meaningful conclusions to be drawn.

## Data availability statement

The original contributions presented in the study are included in the article/**Supplementary Material**. Further inquiries can be directed to the corresponding author.

## Ethics statement

The studies involving humans were approved by the Research Ethics Committee (REC) within the UK Health Departments’ Research Ethics Service. The studies were conducted in accordance with the local legislation and institutional requirements. The participants provided their written informed consent to participate in this study.

## Author contributions

HL: Conceptualization, Data curation, Formal Analysis, Funding acquisition, Investigation, Methodology, Project administration, Resources, Validation, Visualization, Writing – original draft, Writing – review & editing. FM: Data curation, Investigation, Writing – review & editing. GV: Data curation, Investigation, Supervision, Visualization, Writing – review & editing. AL: Data curation, Investigation, Visualization, Writing – review & editing. LS: Data curation, Formal Analysis, Methodology, Visualization, Writing – review & editing. CP: Formal Analysis, Methodology, Writing – review & editing. CFo: Data curation, Writing – review & editing. RB: Data curation, Resources, Writing – review & editing. SK: Data curation, Writing – review & editing. SC: Data curation, Writing – review & editing. AG: Writing – review & editing. CFr: Writing – review & editing. SR: Writing – review & editing. NS: Writing – review & editing. IC: Writing – review & editing. DC: Supervision, Writing – review & editing. NV: Conceptualization, Funding acquisition, Methodology, Project administration, Resources, Supervision, Visualization, Writing – review & editing.
