# Supplementary material for: MicroRNAs as biomarkers for trastuzumab-based therapy in HER2-positive advanced oesophago-gastric cancer patients
Source: Front Oncol. 2023 Nov 29;13:1258365. doi: 10.3389/fonc.2023.1258365 (PMC10718572; doi:10.3389/fonc.2023.1258365)
Supplement: Supplementary file 1 [file DataSheet_1.docx]

**Supplementary methods**

**RNA Sequencing**

Transient inhibition of miR-148a-3p versus DharmaFECT2 control was performed in the HER2 positive NCI-N87 and OACP4C oesphago-gastric cancer cell lines and RNA Sequencing was performed Arraystar Inc (Arraystar Inc, Rockville, Maryland, USA).

**Supplementary Results**

**Large-scale microRNA inhibitor screen results**

Results from the high-throughput large-scale RNA interference screen using a library of LNA™ miR-inhibitors identified 59 statistically significant miRs in the HER2 positive oesophago-gastric cancer NCI-N87 cell line: miR-636, miR-1296-5p, miR-1249-3p, miR-197-3p, miR-1234-3p, miR-664a-3p, miR-3180-5p, miR-133b, miR-1825, miR-1224-3p, miR-1260a, miR-361-3p, miR1247-5p, miR-625-3p, miR-491-3p, miR-326, miR-877-3p, miR-486-5p, miR-296-5p, miR-324-3p, miR-483-3p, miR-1225-3p, miR-767-5p, miR-223-3p, miR-615-5p, miR-28-3p, miR-362-5p, miR-365a-3p, miR-566, miR-615-3p, miR-331-3p, miR-26a-1-3p, miR-551b-3p, miR-374b-5p, miR-328-3p, miR-7-5p, miR-518e-3p, miR-579-3p, miR-887-3p, miR-744-5p, miR-518a-3p, miR-155-3p, miR-10a-5p, miR-1913, miR-708-5p, miR-543, miR-548a-3p, let-7e-3p, miR-519d-3p, miR-500a-5p, miR-148a-3p, miR-361-5p, miR-875-5p, miR-373-3p and miR-488-3p (in order of greatest to least effect on cell viability when inhibited) (**Supplementary** **Figure 1 (A)**), and 37 statistically significant miRs in the HER2 negative oesophago-gastric cancer FLO-1 cell line: miR-1249-3p, miR-3180-5p, miR-1296-5p, miR-361-3p, miR-664, miR-197-3p, miR-223-3p, miR-484, miR-1825, miR-296-5p, miR-1237-3p, miR-1234-3p, miR486-5p, miR-615-5p, miR-1247-5p, miR-566, miR-133b, miR-491, miR-133a-3p, miR-129-3p, miR-93-3p, let-7e-3p, miR-374b-5p, miR-191-5p, miR-199a-5p, miR-1910-5p, miR-454, miR-10a-5p, miR-21-5p, let-7a-2-3p, miR-9-5p, miR-1245a, miR-1307-3p, miR-3186-5p and miR-635 (in order of greatest to least effect on cell viability when inhibited) (**Supplementary Figure 1 (B)**) when treated with chemotherapy and trastuzumab.

We identified 236 statistically significant miRs in the HER2-positive oesophago-gastric cancer NCI-N87 cell line (Supplementary **Figure 1 (C)**) when treated with single-agent trastuzumab in a single-replicate. In the HER2-positive oesophago-gastric cancer NCI-N87 cell line, inhibition of miR-1236-3p, miR-1296-5p, miR-1249-3p, and miR-197-3p resulted in the greatest fall in cell viability when treated with chemotherapy and trastuzumab (**Supplementary Figure 1 (C)**).

Supplementary **Figure 1 (D)** is a scatter plot showing the data distribution of significant hits in each of the large-scale microRNA-inhibitor screens performed (average cell viability (CV) in the NCI-N87 and FLO-1 trastuzumab+chemotherapy screens and in the NCI-N87 single-agent trastuzumab screen).

**Validation results in cell lines**

Inhibition of miR-148a-3p, miR-7-5p, miR-331-3p, miR-365a-3p and miR-28-3p do not lead to statistically significant differences in cell viability as compared to controls in the HER2 positive oesophago-gastric cell line NCI-N87 when treated with trastuzumab and chemotherapy (**Supplementary Figure 2 (A)**) or single-agent trastuzumab (**Supplementary Figure 2 (B)**). For trastuzumab and chemotherapy (**Supplementary Figure 2 (A)**), miR-148a-3p inhibition caused Δ 67% (p=0.17), miR-7-5p inhibition caused Δ 57% (p=0.75), miR-365a-3p inhibition caused Δ 57% (p=0.80), miR-331-3p inhibition caused Δ 45% (p=0.21), miR-1260a inhibition caused Δ 18% and miR- 28-3p inhibition caused Δ 59% (p=0.49). MiR-1260a inhibition causes direct cytotoxicity to NCI-N87 cells and also led to statistically significant differences in cell viability as compared to controls: viability was significantly reduced even for the ‘no drug’ condition and Δ was 18%, (p <0.005). For single-agent trastuzumab (**Supplementary Figure 2 (B)**), miR-148a-3p inhibition caused Δ 46% miR-148a-3p (p = 0.39), miR-7-5p inhibition caused Δ 34% (p = 0.79), miR-365a-3p inhibition caused Δ 28% (p = 0.42), miR-331-3p caused Δ18% (p = 0.15), miR-28-3p inhibition caused Δ 19% (p = 0.079). MiR-1260a inhibition again appeared directly cytotoxic and Δ was 6% (p = <.0.05).

Based on the fact that inhibition of miR-148a-3p caused the largest difference Δ in the HER2-positive oesophago-gastric cell line NCI-N87, it was further studied in the HER2-positive oesophago-gastric cell line OACP4C treated with trastuzumab and chemotherapy (**Supplementary Figure 2 (C)**) or single-agent trastuzumab (**Supplementary Figure 2 (D)**). There was no significant change in cell viability as compared to controls for miR-148a-3p inhibition (n=3) in the HER2-positive oesophago-gastric cell line OACP4C treated with trastuzumab and chemotherapy (**Supplementary Figure 2 (C)**) or single-agent trastuzumab (**Supplementary Figure 2 (D)**). MiR-148a-3p inhibition caused Δ 50% (n=3) in HER2 positive oesophago-gastric cancer OACP4C cells treated with trastuzumab and chemotherapy (**Supplementary Figure 2 (C)**) and Δ 11% for HER2 positive oesophago-gastric cancer OACP4C cells treated with single-agent trastuzumab (**Supplementary Figure 2 (D)).**

Images of HER2-positive oesophago-gastric cancer NCI-N87 cell lines following inhibition of miR-148a-3p, miR-7-5p, miR-331-3p, miR-365a-3p, miR-28-3p and miR-1260a are shown in **Supplementary Figure 2 (E)**. There was visual evidence of cell death 24 hours after inhibition of miR-1260a (before trastuzumab or chemotherapy is added), indicating inhibition of miR-1260a results in direct cytotoxicity. All cell lines demonstrated increased cell death 96 hours after drug treatment.

RNA sequencing did not identify any pathway effect of miR-148a-3p inhibition in HER2-positive oesophago-gastric cancer cell lines NCI-N87 and OACP4C. The individual genes that are upregulated or downregulated are shown in **Supplementary Figure 2 (F).** There are no concordant upregulated genes between the two HER2-positive oesophago-gastric cancer cell lines NCI-N87 and OACP4C. (**Supplementary Figure 2 (F)**).

One of the miRs identified in the large-scale miR inhibitor screen, miR-1260a, demonstrated the interesting characteristic of directly causing cell death when inhibited, regardless of drug therapy. Further translational and mechanistic studies into miR-1260a might be of interest in HER2-positive (and potentially also HER2 negative) oesophago-gastric cancer, as this miR may hold therapeutic potential.

**Supplementary Figure Captions:**

**Supplementary Figure 1**: Large-scale microRNA inhibitor screen results (A) Bar chart showing significant microRNAs (59) in NCI-N87 trastuzumab + chemotherapy screen (microRNAs that, when inhibited, caused average cell viability to decrease to less than 0.6, p<0.001) relative to control. Results are listed according to average cell viability. N=3. Bars represent average cell viability and error bars represent standard deviation. (B) Bar chart showing the 37 significant microRNAs in FLO-1 trastuzumab + chemotherapy screen (microRNAs that, when inhibited, caused average CV<0.6, p<0.001 relative to control). Results are listed according to cell viability. N=3. Bars represent average cell viability and error bars represent standard deviation. (C) Bar chart showing the 236 microRNAs that were significant in the single-agent trastuzumab screen in the NCI-N87 cell line. Significant microRNAs were those that, when inhibited, caused average CV<0.6. Results are listed in order of cell viability. N=1 (D) Data distribution scatter plots for trastuzumab + chemotherapy screens in NCI-N87 and FLO-1 cell lines (CV<0.6 + *p*<0.001) and single-agent trastuzumab screen in NCI-N87 cell line (CV<0.6). N=3 for the trastuzumab and chemotherapy screens and n=1 for the single-agent trastuzumab screen.

**Supplementary Figure 2: Cell line viability results and RNA sequencing results.** (A) and (B) Bar chart showing effect on cell viability with inhibition of miR-148a-3p, miR-7-5p, miR-331-3p, miR-365a-3p, miR-28-3p and miR-1260a (n=3) in the HER2 positive oesophago-gastric cell line NCI-N87 treated with trastuzumab and chemotherapy (A) or single-agent trastuzumab (B). Controls were no miR (DF2 only) and LNA negative control B. Additional controls in panel B were no miR no DF2, and siTOX. Error bars represent standard deviations. Cell viability is normalised to LNA negative control B ‘no drug’. Unpaired t-test statistical analysis was performed to analyse the effect of miR inhibitors as compared to LNA neg B and p values are included on the graph. (C) and (D) Bar chart showing effect on cell viability with inhibition of miR-148a-3p (n=3) in the HER2 positive oesophago-gastric cell line OACP4C treated with trastuzumab and chemotherapy (C) or single-agent trastuzumab (D). Negative controls were no miR no DF2, no miR (DF2 only, LNA neg B). Positive control was siTOX. The error bars represent the standard deviations. (E) Images of HER2 positive oesophago-gastric cell line NCI-N87 cells 10X magnification at two separate timepoints: pre-drug (24 hours post-transfection) and post-drug (96 hours after the addition of trastuzumab, cisplatin and 5FU). The scale bar is 100μM. (F) Heatmap showing genes that are significantly upregulated and downregulated in the HER2 positive oesophago-gastric cell lines NCI-N87 and OACP4C when miR-148a-3p is inhibited. Cut-off for log2FC>=0.585, p-value cut-off <=0.05, q-value cut-off <=1.00. MAP1S, ZBTB9 and AJUBA are downregulated in the OACP4C cell line but upregulated in the NCI-N87 cell line.

**Supplementary Figure 3:** Results from the Phase 2 PLATFORM trial sub-study for advanced HER2 positive oesophago-gastric cancer patients. (A) Kaplan-Meier curve of Overall survival (OS) miR-148a-3p LOW versus HIGH. Median OS in the miR-148a-3p low group was 12.8 months (95% CI: 7.8-17.0) as compared to 12.4 months (95% CI: 9.0-20.0) in the miR-148a-3p high group, HR 0.98 (95% CI: 0.57-1.66), n=62, p=0.933. (B) Kaplan-Meier curve of Progression Free Survival (PFS) miR-148a-3p LOW versus HIGH. Median PFS in the miR-148a-3p low group was 6.7 months (95% CI: 4.6-8.4) as compared to 7.2 months (95% CI: 4.3-7.9) in the miR-148a-3p high group, HR 1.08 (95% CI: 0.65-1.81), n=62, p=0.759. (C) Progression free rates (PFR) for miR-148a-3p LOW versus HIGH. PFR at 3 months were 53% (95% CI: 29.2 – 76.7) in the miR-148a-3p low group compared to 43% (95% CI: 16.9-68.8) in the miR-148a-3p high group (OR 0.67 (95% CI: 0.2-2.8), n=31, p=0.577.
