# Supplementary figures and images for: MicroRNAs as biomarkers for trastuzumab-based therapy in HER2-positive advanced oesophago-gastric cancer patients
Source: Front Oncol. 2023 Nov 29;13:1258365. doi: 10.3389/fonc.2023.1258365 (PMC10718572; doi:10.3389/fonc.2023.1258365)

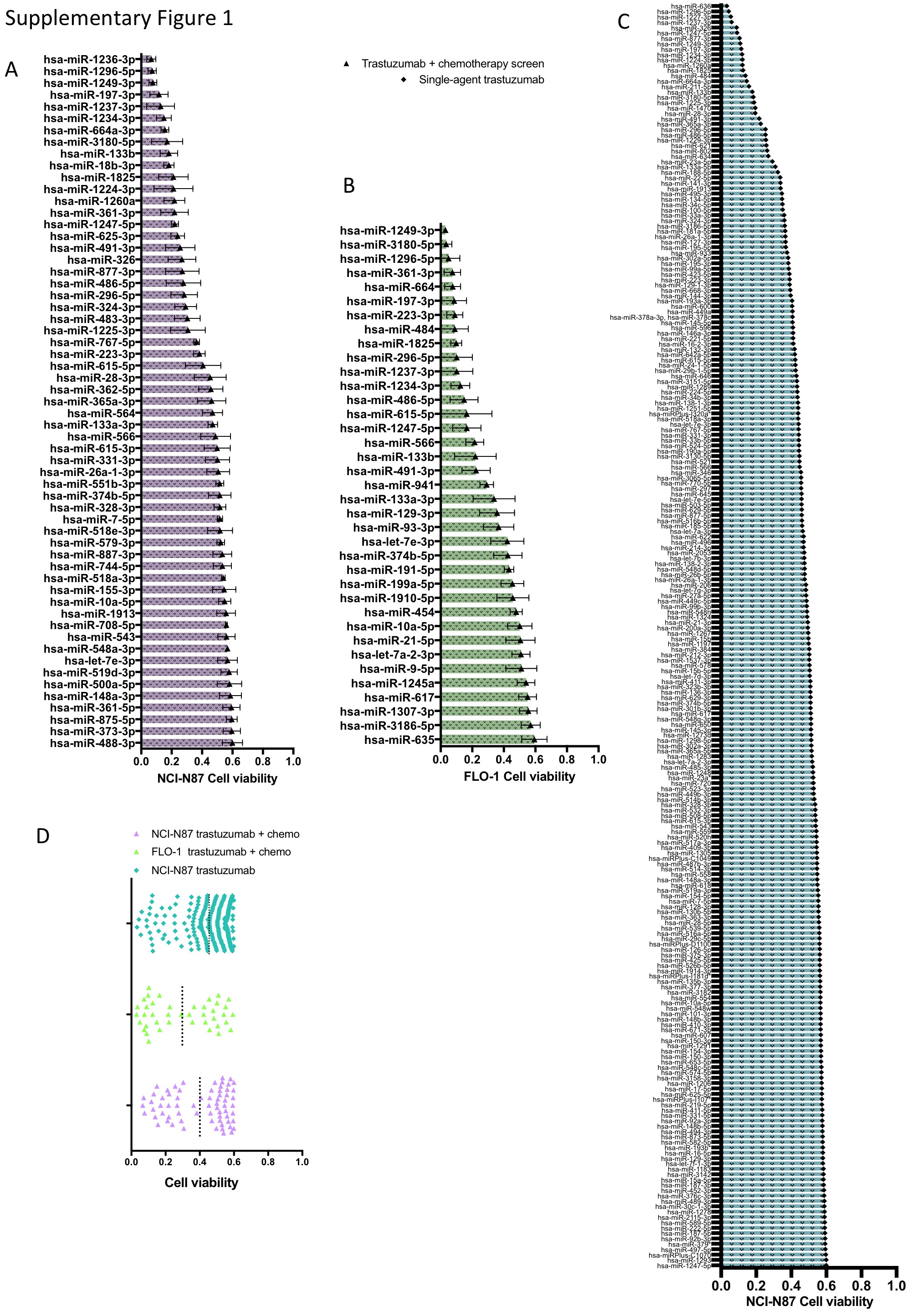

Supplement: Supplementary file 2 [file Image_1.jpeg]

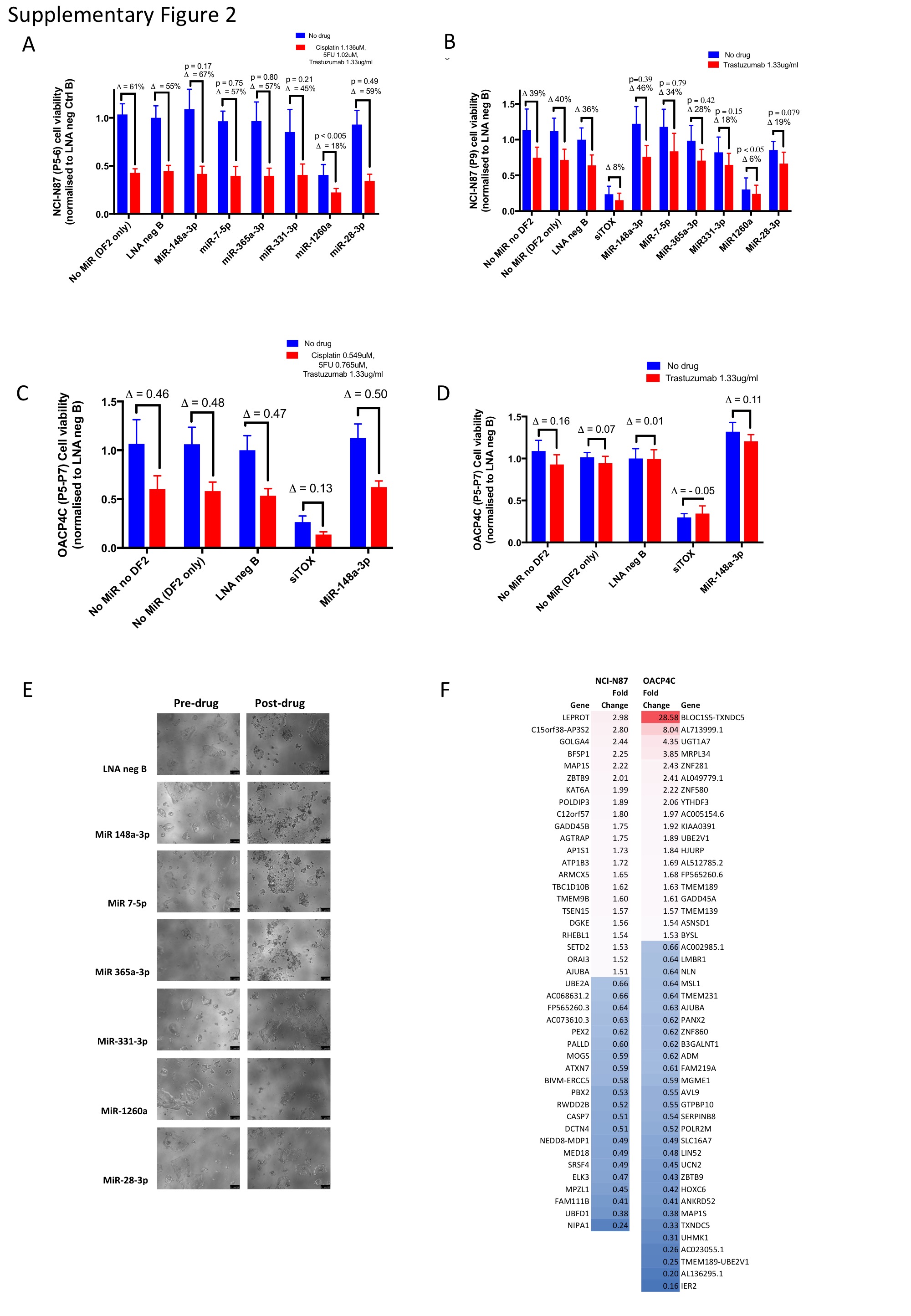

Supplement: Supplementary file 3 [file Image_2.jpeg]

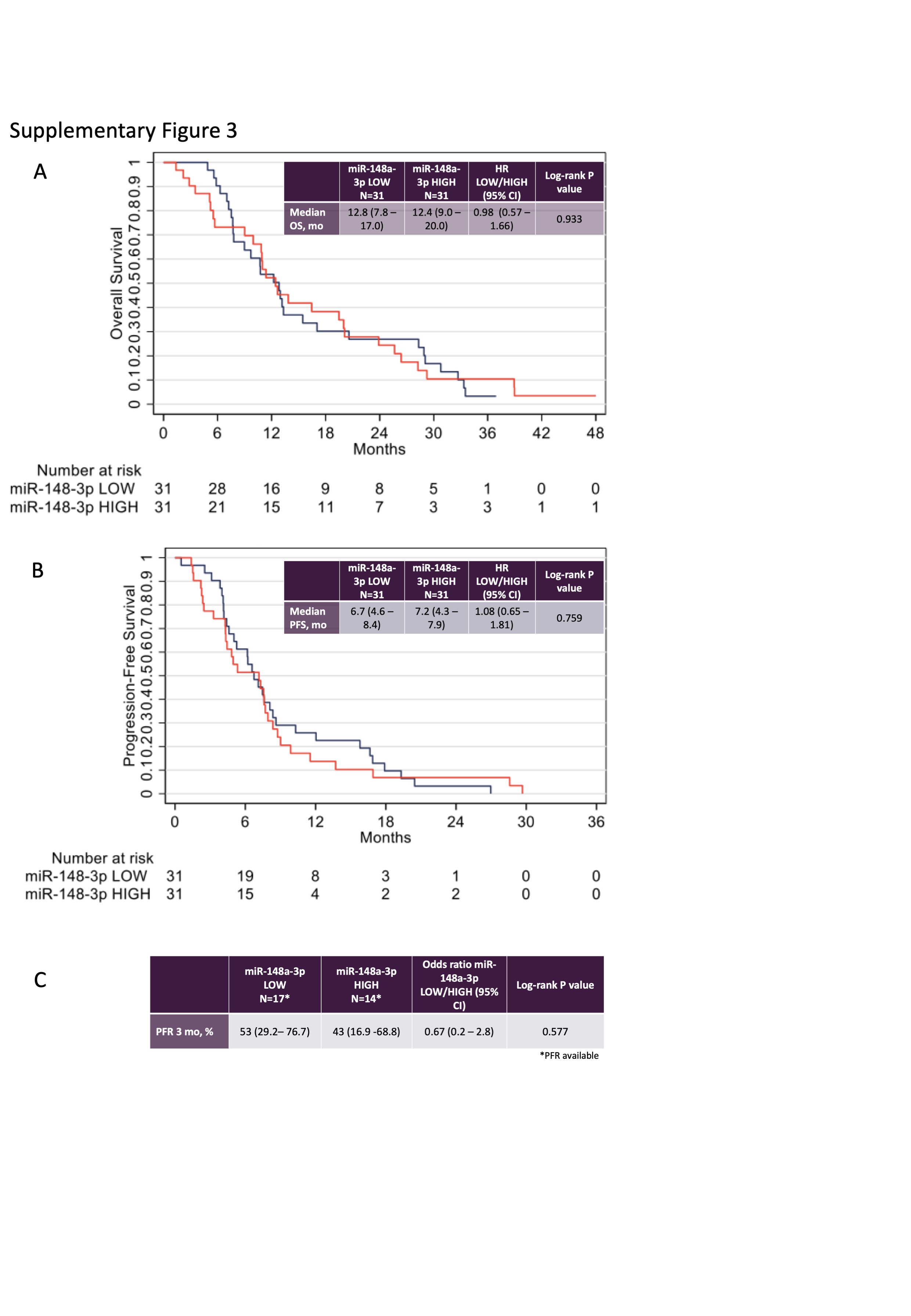

Supplement: Supplementary file 4 [file Image_3.jpeg]
